# Tool-Use Training in a Species of Rodent: The Emergence of an Optimal Motor Strategy and Functional Understanding

**DOI:** 10.1371/journal.pone.0001860

**Published:** 2008-03-26

**Authors:** Kazuo Okanoya, Naoko Tokimoto, Noriko Kumazawa, Sayaka Hihara, Atsushi Iriki

**Affiliations:** 1 Laboratory for Biolinguistics, Brain Science Institute, RIKEN, Saitama, Japan; 2 Laboratory for Symbolic Cognitive Development, Brain Science Institute, RIKEN, Saitama, Japan; Università di Parma, Italy

## Abstract

**Background:**

Tool use is defined as the manipulation of an inanimate object to change the position or form of a separate object. The expansion of cognitive niches and tool-use capabilities probably stimulated each other in hominid evolution. To understand the causes of cognitive expansion in humans, we need to know the behavioral and neural basis of tool use. Although a wide range of animals exhibit tool use in nature, most studies have focused on primates and birds on behavioral or psychological levels and did not directly address questions of which neural modifications contributed to the emergence of tool use. To investigate such questions, an animal model suitable for cellular and molecular manipulations is needed.

**Methodology/Principal Findings:**

We demonstrated for the first time that rodents can be trained to use tools. Through a step-by-step training procedure, we trained degus (*Octodon degus*) to use a rake-like tool with their forelimbs to retrieve otherwise out-of-reach rewards. Eventually, they mastered effective use of the tool, moving it in an elegant trajectory. After the degus were well trained, probe tests that examined whether they showed functional understanding of the tool were performed. Degus did not hesitate to use tools of different size, colors, and shapes, but were reluctant to use the tool with a raised nonfunctional blade. Thus, degus understood the functional and physical properties of the tool after extensive training.

**Conclusions/Significance:**

Our findings suggest that tool use is not a specific faculty resulting from higher intelligence, but is a specific combination of more general cognitive faculties. Studying the brains and behaviors of trained rodents can provide insights into how higher cognitive functions might be broken down into more general faculties, and also what cellular and molecular mechanisms are involved in the emergence of such cognitive functions.

## Introduction

Through tool use, hominids expanded the range of available biological and physical resources and changed their own adaptive niches, resulting in further expansion of cognitive capacities [1], [2]. Here, tool use is defined as manipulation of an inanimate object to change the position or form of a separate object [3]. Simple forms of tool use are observed in a wide range of animals [3], but to examine the functional and causal understanding of tools, tool-use behavior has been extensively studied only in nonhuman primates [4]–[7] and birds [8], [9]. While these studies are important for examining behavioral and evolutionary hypotheses regarding the origin of human tool use, tool-use behavior should result in modifications of brain architecture and such modifications should again affect the way tool-use behavior is organized. Thus, not only behavioral, but also the neural and molecular bases of tool-use behavior, should be investigated to clarify the causes of cognitive expansion in humans. We need a practical animal model with which to efficiently explore the neural and molecular bases of tool use.

In contrast to naturalistic tool use in the wild, tool-use training in captive environments not only provides insights into the cognitive potential of animals [10], [11] but may also provide a neurobiological platform for fruitful extrapolations about the higher mental faculties of humans [12], [13]. Although such a non-naturalistic approach may have a drawback in that ecological and evolutionary relevance could be tenuous, it may alternatively provide powerful probes into the neurobiology of advanced cognitive functions under precise experimental control. This approach has been successful in a series of studies in macaques: tool-use training resulted in spontaneous refinement of motor trajectory [10] and the spontaneous and rapid application of meta-tools [14]. Moreover, it was accompanied by gene expression and circuit reorganization in the intraparietal cortex, which led to the emergence of novel functional connections with the temporoparietal and prefrontal areas [15].

As such, macaque monkeys have been the only animal models for neurobiological investigations based on the assumption that their phylogenic proximity to humans might aid successful extrapolation of findings [16], [17]. However, here we provide a rodent model for tool use, which should dramatically widen the range of experimental manipulation. Although some research has examined natural tool use in rodents [18], [19], this is the first study of controlled tool-use training. The species we used for this study is the degu. We decided to test degus' capacity for tool use because of the following ensemble of evidence, all of which implied the superb manual dexterity and good eye–hand coordination essential for tool use. Degus are highly social, diurnal rodents [20] that use visual, auditory, and olfactory cues in their social communication [21]. The openings of degu burrows are mounds adorned with piles of sticks, stones, and cow dung [22]. Captive degus engage in the spontaneous construction of nesting cups, which has been rarely observed in primates [23]. Moreover, degus engage in several types of nonfunctional “play”-type behavior among nest mates [24] and have well developed prefrontal areas sensitive to early social deprivation [25]. This evidence implies a high level of curiosity in this species, which makes it suitable for cooperative experimenter–subject relations, as well as the behavioral flexibility required for tool-use training.

## Results

**Figure 1:**
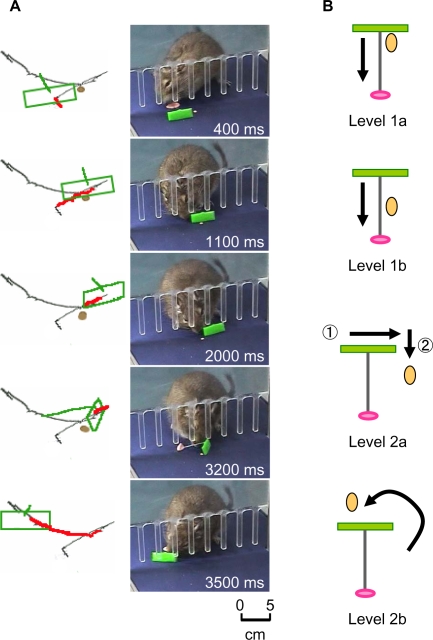
Performance example of tool use and levels of training. A. An example of the performance by one degu at Level 2a. Right column: Video frames depicting representative epochs during a single attempt at food retrieval using a rake tool. In the beginning, the degu failed to draw in the sunflower seed (1,100 ms). The animal then adjusted the position of the tool carefully after it observed the position of the food (2,000 ms) and pulled in the reward successfully (3,200 ms). Left column: Trajectories of the tip of the tool between the previous and following frames (red lines) are superimposed on the trajectory during the entire course of retrieval (gray lines, identical for all frames). Orientation and position of the rake (green squares and bars) and the location of food (orange dots) are also shown (traced from the photographs in the right column). B. Levels of training. At Level 1a, the animal can simply pull the tool toward itself. At Level 1b, the distance to pull is increased. At Level 2a, the animal has to make a lateral movement 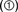 before pulling the tool toward itself 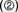. At Level 2b, the animal has to place the tool beyond the reward before pulling.

We trained five adult degus to use a rake-like tool to retrieve a distant food reward. The tool was a T-shaped rake that consisted of a wire shaft and a rectangular plate attached perpendicularly at the end. Training was conducted in a chamber that had a fence separating the animal and the food reinforcement. A step-by-step training program similar to that used for macaques [10], [11] was established and degus were trained accordingly (supplementary video 1, Movie S1). A trial began when a food item was placed outside the fence and out of the animal's reach, followed by the tool being placed outside the fence with its grip facing the animal. The degu was then allowed to manipulate the tool to retrieve the food (Fig. 1A). Training was conducted at two diff**e**rent levels (Levels 1 and 2), each of which consisted of two different sublevels (“a” and “b”) of difficulty according to the relative position of the tool and the reinforcement (Fig. 1B). The distance between the tool and the reward was initially very close (Level 1a) and then was extended gradually (Level 1b; Movie S2). After the animal learned this task, the reward began to be placed at the side of the tool so that the animal had to move the tool laterally before pulling it in (Level 2a; Movie S3). Finally, the food was placed beyond the tool so that the animal had to first push the tool beyond the food, move it laterally, and then pull on it to get the food (Level 2b; Movie S4).

**Figure 2:**
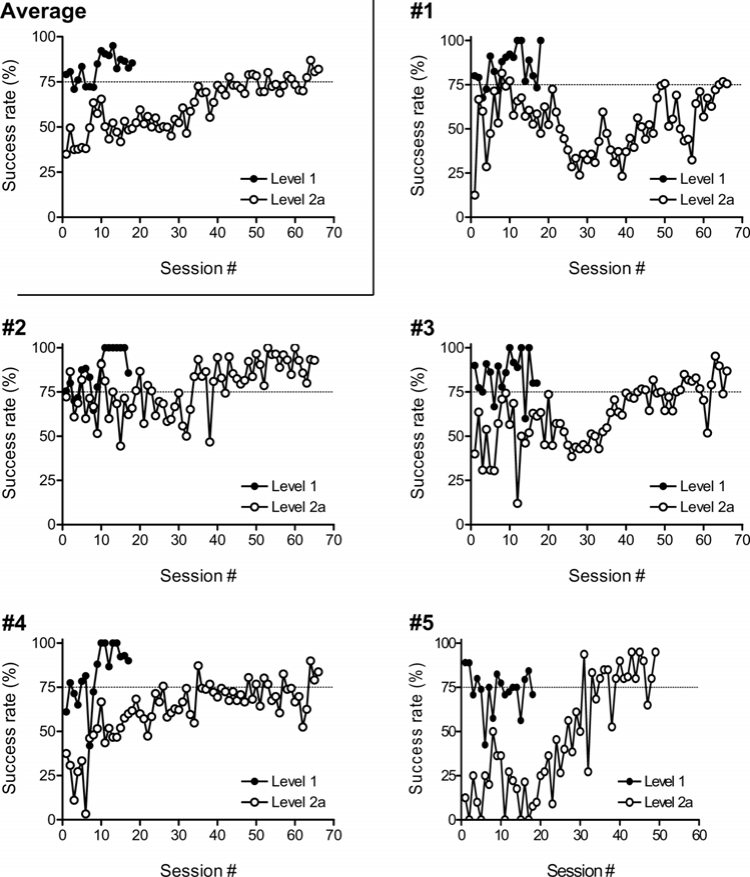
The average and individual learning curves. Left top: The average success rate (in percent; *y*-axis) plotted by session (*x*-axis) for Level 1a (first session only) and Level 1b (2^nd^–18^th^ sessions) (filled symbols) and Level 2a (open symbols). Sessions were numbered independently for Levels 1 and 2 and aligned at the origin of the abscissa because sessions for later Level 1 and early Level 2—easy and difficult sessions, respectively—were intermingled to keep the animals motivated. Note that in practice, Level 2a training contained trials that resulted in Level 2b difficulty after the first retrieval attempt failed, leaving the food behind the tool. Each data point represents the average of the five animals. The rest of the graphs: Learning curves of individual animals (#1–#5). The success rate (in percent; *y*-axis) is plotted by session (*x*-axis) for Level 1a (first session), Level 1b (2^nd^–20^th^ sessions) (filled symbols), and Level 2a (open symbols) for each of the five animals. Level 1a training was given only for one session. For Levels 1b and 2a, the criterion for success was 75% or higher in three consecutive sessions. After the criterion was satisfied, some animals received additional training. Sessions are numbered independently for Levels 1 and 2, and are aligned at the origin of the abscissa.

**Figure 3:**
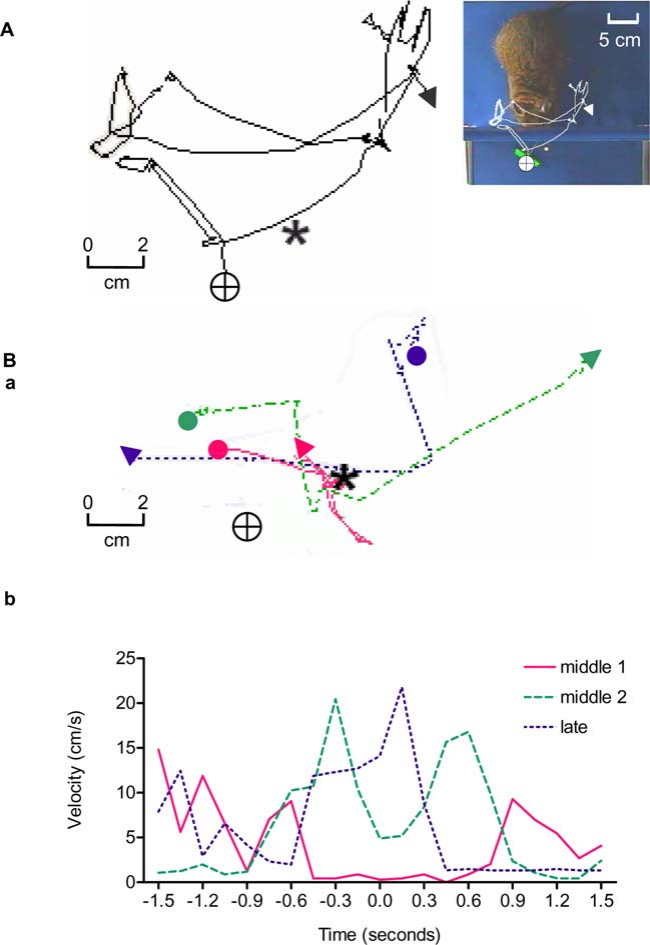
Trajectory and velocity of the tip of the tool. The circled crosses in A and B-a represent the center of the tool blade, and the asterisks indicate the position of the reward at the beginning of a trial. Filled circles denote the initial points and triangles represent the endpoints of the tool head. A: Representative trajectory of the tool head before extensive training. Right: A photograph of the top view. Left: Trajectory drawn from multiple video frames. The degu waved the tool for a while around the reward but failed to obtain the reinforcement in this trial. B: Representative examples of trajectories (a) and corresponding velocity profiles (b) of the tool head chosen from an early-middle phase of training (middle 1; red line), a late-middle phase (middle 2; broken green line), and a late phase (dotted blue line) during Level 2a training in one degu. In B-a, time zero indicates the time when the degu changed the angle of the rake upon approaching the reward. In the early-middle phase, the velocity of the tool was generally slow and became particularly slow at around time zero, presumably because the degu took time to adjust the angle of the tool relative to the food. In the late-middle phase, the velocity peaked at −0.4 s and 0.6 s and decreased after the degu changed the angle of the tool at time zero. In the late phase, the velocity peak of the tool came at around time zero. Movie clips from which the above data were obtained are shown in Movie S5 [Supplementary-material pone.0001860.s006] [Supplementary-material pone.0001860.s007]-8.

Figure 2 illustrates the averaged and individual time course of five degus that learned to use the tool with their forelimbs (criterion for acquisition: ≥75% for three successive sessions). Level 1a training was easy for them and all five animals completed it within one session (filled symbol, first session). In contrast, the number of sessions required to successfully reach Level 1b (filled symbols, 2^nd^ session and after) varied from 6 to 11 depending on the individual. In the initial phase of Level 2a (open symbols), most animals did not retry after the first (failed) attempt to pull in the food, whereupon the difficulty level was converted to Level 2b. The success rate then dropped to about 40% (Fig. 2; around the 15^th^ sessions). As the training continued in Level 2a, the animals began to hold the tool for a longer time after an initial failure. This longer, more secure tool holding was followed by the emergence of new characteristic behavioral patterns shown in Figure 3. The degus began to move the rake back and forth and around the reward, pushing the tool or wiggling it horizontally (Fig. 3A, Movie S5). With this shift in behavior, success rates again began to increase (Fig. 2; around the 30^th^ sessions). At this early phase (20^th^–35^th^ sessions), the animals began to stare at the food rather than the tip of the tool as they had at the very beginning of the training.

During the middle phase (35^th^–50^th^ sessions) of Level 2a training, all subjects learned to control the rake with two distinct motions: first, moving the tool horizontally toward the food, and second, pulling it vertically toward themselves (red line and broken green line in Fig. 3B-ab, Movie S6 and Movie S7). These two motions could be clearly identified as two peaks in their velocity profiles, as depicted in Figure 3Bb. The time separation between these two peaks shortened as their skill improved, suggesting increasing efficiency of usage. All five animals successfully advanced to Level 2b training after an average of 57 sessions (range 36–67 sessions). In the late phase (about the >50^th^ session) of the training, in three of the five animals, a very smooth trajectory consisting of continuous motions exhibiting a single velocity peak (dotted blue line, Fig. 3Bb, Movie S8) occasionally appeared.

**Figure 4:**
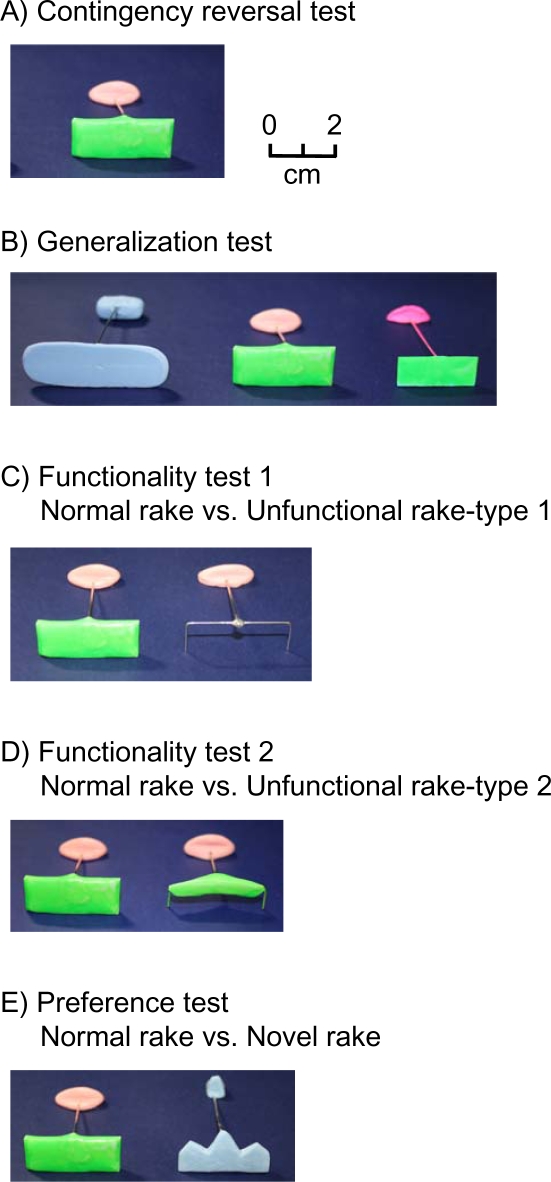
Varieties of rake (left photographs) used in the probe tests (summary of results in table 1) to examine conceptual understandings of tool use. A) Reversed contingency test (see also Movie S9 for the behavior). The same tool as used in training was used in this test. B) Generalization test (Movie S10). New tools are shown on the left and right; performance on these new tools was tested in ten trials each on four animals in random order. C) Functionality test 1 (Movie S11). The normal tool is on the left and a nonfunctional tool without a blade is shown on the right. D) Functionality test 2 (Movie S12). Another nonfunctional tool, which has a raised blade, is shown on the right. E) Preference test (Movie S13). A functional but unusual-looking tool is shown on the right. The animals did not hesitate to use this tool. In test A, the same tool as that in training was used.

Four of the five animals were further tested for their conceptual understanding of the tool in a series of probe tests (Fig. 4, Table 1) [5], [6]. Under the first condition (A), the familiar sequence of stimulus presentation was reversed—tool first, food second—to see if it would disrupt the animals' acquired skill. Second (B), two new tools that differed in color, shape, or size were presented to test whether the degus could generalize their acquired tool-use facility. Third (C, D), two tools—the functional one that had been used for the training and a nonfunctional one that had the plate of the tool raised by wire so that food could not be retrieved—were given to the degus in a simultaneous choice test to see which tool they selected first. Finally (E), aversiveness to novelty (i.e., a xenophobic tendency of the animals) was tested in a same choice test by presenting two tools, one that had been used in training and another functional but novel tool. The final condition was added to determine whether the unfamiliarity with the second tool acted as an aversive stimulus.

**Table 1:** Results of the probe tests.

					(%)
Degu No.	#1	#2	#3	#4	Average
A) Contingency reversal test	70	60	90	70	72.5
B) Generalization test	100	100	100	100	100
C) Functionality test 1	70	70	80	70	72.5
D) Functionality test 2	70	60	70	70	67.5
E) Preference test	40	50	70	30	47.5

Ten trials were given for each probe test to each animal. Numbers in the table in tests A and B indicate the percentage of successful performances. In tests C, D, and E, numbers indicate the percentage of selecting the tool used in the training.

Under the first condition (A), when the order of presentation was reversed so that the tool was already available before a new reward was placed, all four subjects manipulated the tool just as in training and their behavior was not disrupted by the reversed contingency (success rate 60–90%) (Movie S9). The degus were not randomly manipulating the tool before the new reward was placed, and they began to manipulate the tool purposefully when a new reward was placed on the platform. Under the second condition (B), the degus showed quick generalization to the new tools (success rate 100% for all animals, Movie S10). Under the third condition (C and D), they selected the functional tool over the nonfunctional one (success rate 60–80%, Movie S11 and Movie S12). Under the final condition (E), the degus showed no sign of aversion to the unfamiliar tool (average 47.5%, Movie S13) when two equally functional tools were presented. Binomial tests comparing performances in each of the probe tests C, D, and E revealed that the four animals as a group selected the functional tool over the nonfunctional one significantly more often (C: p = 0.006; D: p = 0.038) but they chose between two functional tools at random ( E: p = 0.875). In sum, the results of the probe tests suggest that the animals understood the tools' functional properties (i.e., whether the tool could be used to retrieve food) and ignored their irrelevant properties (i.e., color and familiarity, and within certain ranges, shape and size) (Table 1).

## Discussion

Our results show that a species of rodent, the degu, can be trained to manipulate a rake-like tool using the forelimbs to retrieve a distant food reward. After extensive training for the task, degus showed functional understanding of the tool. Together with reports on tool use and its functional understanding in birds [9], [26], [27], our results justify a change in the conventional view of animal intelligence based on phylogenic relations with humans [28]. Our findings suggest a refined view in which the prerequisites for higher cognitive functions, including tool use and its functional understanding, may occur in a wide range of animals; moreover, socio-ecological factors may be more important than phylogenic factors for such functions to evolve [9].

After learning the basic skill of simply pulling the tool, the degus spontaneously devised more flexible, efficient and versatile use of the tool. The number of trials (about 2,500 trials, 35–45 trials per day, average 57 days) required to train degus for tool use was comparable to that required for Japanese macaques (about 2,600 trials, 160–250 trials per day, 13–14 days) in a similar task [10], and to similar criteria for trials to be regarded successful. Moreover, highly trained degus showed similar trajectories of tool manipulation as those seen in Japanese macaques [10]. In the middle phase, lateral movement and pulling consisted of two distinct trajectories with two peak velocities, which as efficiency increased, gradually merged into one smooth, continuous trajectory that minimized the time and energy required to obtain the reward (Fig. 3B). The degus were taking advantage of some of the tool's physical properties such as mass, inertia, and friction. These observations suggest that tool use by degus and Japanese macaques share some of behaviorally common characteristics that may represent a standardized set of cognitive skills necessary for general implementation of tool use.

After extensive training on tool use, probe tests demonstrated that degus gained functional understanding of the tool. The degus ignored irrelevant tool properties such as shape, size, and color while paying attention to its functional attributes in attaining food reinforcement. The results of our probe tests are comparable to those of the vervet monkey [6]. These results can be interpreted to mean that degus attempt mental manipulation of the tool before actually selecting an alternative. These results suggest that through training, degus developed a mental representation of the tool that focused heavily on the functional aspect.

Tool use in macaques led to the expression of immediate early genes and neurotrophic factors in the intraparietal cortex [28] and resulted in intraparietal bimodal neurons (which integrate somatosensory and visual information relating to the hand/forearm) extending their visual receptive fields to include tools as extensions of innate body parts. In the rodent brain, sensory plasticity as demonstrated by gene expression has been detected in the barrel cortex [29] and auditory cortex [30], and motor plasticity after new kinetic training was revealed in the motor cortex [31]. Observing the degree of plasticity in the rodent brain, we can reasonably assume that the present tool-use training in degus could also results in extended representations in parietotemporal areas and newly formed connections between brain areas, including the prefrontal cortex, similar to that observed in the macaque brain. This would comprise an immediate extension of the present study, among other potential neurobiological examinations.

Here we showed that given precise experimental controls, rodents can be trained to perform a complex task. Having established a rodent model for tool use, we can now ask further questions regarding the neurobiology of tool use. Our demonstration of successful training of tool use in rodents should encourage such studies in other species that are amenable to similar environmental, behavioral, genetic, and physiological manipulations. Which types of neural plasticity, perhaps including adult neurogenesis [32], are involved in the emergence of acquired purposive behavior? How does hand–eye dexterity develop through training? Does the rodent brain have a mirror neuron system, and if so, how might this enhance their general cognition? These are all questions raised by the current rodent model. By shaping a challenging behavior that involves combinations of several brain areas not normally connected by ecological pressures, we can modify existing brain circuitry in the animals [15]. Such “constructive” neuroscience based on the enhancement of animals could be a new paradigm for tackling many otherwise intractable puzzles of human intelligence.

## Materials and Methods

### Animals and their maintenance

Four male (10-month-old) and one female (5-month-old) degus (weight 180–240 g) from three litters were tested. Initially, we selected two animals randomly from each of three families. Thus, we used a total of six animals, two females and four males. However, while it performed as well as the other animals, we had to discard data from one female because we applied an incorrect criterion for successful training (two successful sessions in a row instead of three). No animals were excluded from the experiment because of poor performance. Of the five animals used in the final data analyses, the female was animal #5 in Figure 2 and this individual did not differ from the other animals in terms of performance. The animals were reared normally with their parents and littermates. Three to six animals were kept in a large cage (45×70×30 cm) that contained a metal running wheel, a wooden sleeping den and tunnel, nesting materials, a food cup, and a water supplier. Food pellets and water were freely available, and the animals were not deprived of food prior to the experiments. The degus were kept in an animal room at the RIKEN Brain Science Institute under a fixed 12-h day, 12-h night cycle, with temperature around 22°C and relative humidity around 50%. This experiment was approved by the RIKEN Brain Science Institute animal experiment committee #H18-2B012 and complied with the institutional regulations.

### Apparatus

**Figure 5:**
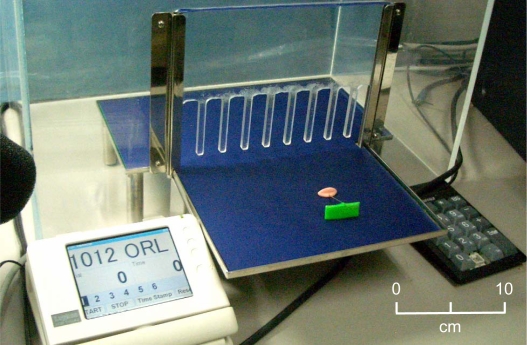
The experimental platform (middle), the TFT monitor for event display (left), and the numeric pad for event recording (right). The scale bar is inserted only as a rough reference because the photo is tilted. The degu was placed behind the fence and the experimenter sat facing the animal from outside the enclosure.

Training was carried out on a training platform made of acrylic board (Fig. 5). The platform consisted of a training stage (20×30×7 cm) and a transparent fence (45×55×0.5 cm) that separated the animal from the area where the food rewards and tools were placed. The fence was placed 1.5 cm above the training stage so that the degu could reach through with its forelimb and grab the tool in its hand, but otherwise no restrictions or impositions were placed on the animal's mobility or actions. Each bar of the fence was 0.5 cm wide and bars were 1.5 cm apart. The training platform was placed on a table set inside a sound-isolation room and the experimenter faced the degu across the table. The rake-like tool was T-shaped, with a wire shaft 4 cm long, a rectangular plate (3 cm wide×1 cm high) made of acrylic resin, and a spherical grip 1 cm in diameter. The tool weighed 2.1 g. The tool and food reward were placed outside the fence with the rake handle within the degu's reach.

A 4-inch LCD monitor (Logitech LCMTo42AS) was placed on the left side of the platform, and a numeric pad was placed on the right so that the experimenter could measure the duration of each trial and also could use it to advance the trial. The LCD monitor and the numeric pad were connected to a personal computer that was programmed to function as an event recorder. Each training session was monitored by two cameras (Sony DCR-PC110) at the top and upper-left side of the platform, and recorded with a video recorder (Panasonic VDR-M30). Recordings were analyzed off-line using motion analysis software (Noldus, Ethovision Color Pro).

### Procedures

1. Habituation: Each animal was habituated to the training environment by placing it in the enclosure and giving it a piece of sunflower seed. The animal was then trained to obtain food placed outside the fence using its forearms. Next, the animal was habituated to the rake tool. A reward was given when the animal touched and dragged the rake. Habituation to the tool and the environment was completed in one session lasting around 1 h.

2. Training: Training was carried out by the successive approximation method, modeled on the Japanese macaque's training procedure. We did not use an auditory cue to shape behavior in any phase of training. During each day of training, the degu was put through one session that consisted of 35 to 45 trials. The degus were trained 5 days a week. Each trial was set up by placing a food item on the table beyond the degu's reach, and then was begun by placing the tool on the table while the experimenter simultaneously pressed the numeric pad to mark the start of the trial. The trial came to an end either when the degu successfully obtained the food or when 60 s had elapsed, which was marked by the experimenter pressing the numeric pad. The duration of the trial was defined as the time difference between the two key presses. After the trial, the tool and food (if remaining) were withdrawn from the platform and an intertrial interval of an average of 9 s (range 3–30 s) followed. The food reward in each trial was half a sunflower seed, the degus' favorite treat. One-half of the skinned seed was given as reinforcement for each trial. The degus performed for sunflower seeds without food deprivation.

At the beginning of the training (Level 1a), the reward was placed close to the animal's side of the rake blade (up to 1 cm *behind* the blade and within 0.5 cm from the center of the blade) and the animal was trained to simply pull the tool. The distance between the tool and reward was then extended gradually (Level 1b; within 1.5 cm). After the animal learned this task, we began to place the reward at the side of the tool so that the animal had to move the tool laterally before pulling it in (Level 2a). Finally, the food was placed *beyond* the tool so that the animal had to first push the tool beyond the food, move it laterally, and then pull on it to get the food (Level 2b). In general, advancement through these sublevels occurred during sessions after a success rate over 50% was attained. Five or more successful performances resulted in advancement to Level 2b, while three failures in a row resulted in retraining under Level 2a.

In Level 1a, the degu could simply pull the tool straight toward itself since the food was placed adjacent to the near side of the plate (Fig. 1B). In Level 1b, the distance between the food and the plate was extended (2^nd^ panel), but the degu could still obtain the food by carefully pulling the tool straight toward itself (Movie S2). In Level 2a, the food was displaced horizontally from the plate, requiring the degu to make a lateral movement (3^rd^ panel, 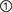) before pulling the tool toward itself (3^rd^ panel, 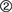, Movie S3). In Level 2b, the reward was placed beyond the plate (4^th^ panel, Movie S4), thus requiring the degu to swivel and push the tool beyond the reward and then adjust the relative position between the reward and the plate before pulling it toward itself. In practice, Level 2a and Level 2b were not clearly distinguishable because when an attempt to pull the reward failed in Level 2a training, the following trial had a Level 2b setting. At this final level, advanced behavioral planning was required for efficient retrieval of the food reward.

In the initial stage of training, after the degu pulled the tool once without success, it often hit the tool by the nose from several directions without further trying to retrieve the reward with the tool. After several failures, it often jumped off the platform or bit the fence. These behaviors suggest that the degu had no understanding of the function of the tool. In the middle stage of training, the degu gradually came to retry efforts several times after an initial failure to retrieve the reward. The degu came to examine the relative position of the food and the tool, without continuing to hold or manipulate the tool. It began to shake the tool to the left and right, and then gradually learned to push and pull the tool. These behaviors suggest that the degu began to appreciate the function of the tool. In the final stage of training, the degu always paid a great deal attention to the relative position of the tool and the reward. At this stage, it often pulled the tool toward itself presumably to gain control over the tool before it tried to retrieve the food. At this stage, the degu tended not to abandon the trial after an initial failure, and continued retrying until it gained the reward.

3. Probe tests: After the tool-use training, the following probe tests were administered to examine the degus' functional understanding of the tool (Fig 4, Table 1). We ran each animal through each probe test ten times. Probe tests consisted of the following: A) Contingency reversal test (Movie S9), B) Generalization test (Movie S10), C) Functionality test 1 (Movie S11), D) Functionality test 2 (Movie S12), and E) Preference test (Movie S13). For more details, see Figure 4 and Table 1. We restricted the number of trials for each probe test to be ten to avoid loss of stimulus control over the tool and also to avoid giving a new set of trainings to the probe tools. For tests C, D, and E we performed statistical tests to examine animals' tendency to select a particular tool. Since the number of probe trials was small, we performed binomial tests on the pooled data of four animals (sums up to 40 trials) and tested whether the animals as a group selected one of the tools significantly more often.

## Supporting Information

Movie S1 Video clip depicting Degus' typical tool-use behavior, corresponding to Fig. 1A.
(2.40 MB MPG)

Movie S2 Video clip depicting Level 1b tool-use behavior presented at half speed (15 frames/sec; normal speed is 30 frames/sec). The degu pulled the tool straight toward itself. (see also Fig. 1B)
(2.44 MB MPG)

Movie S3 Level 2a behavior shown in the same format as Sv2. In 0920OLR, the degu carefully adjusted the direction of the tool before pulling. In 1010G3R, the degu pulled the tool diagonally with smooth movement (see also Fig. 1B).
(4.10 MB MPG)

Movie S4 Two slow-motion clips representing Level 2b behavior. Although the experimenter initially set up at Level 2a conditions, the level of difficulty immediately became that of Level 2b, since the degu pulled the tool toward itself past the food. In the first clip (trial 45) the degu made a clockwise circular movement to get the reward. In the second clip (trial 46) the degu made a counterclockwise movement (see also Fig. 1B).
(8.17 MB MPG)

Movie S5 Level 2a, initial phase. The degu wiggled the tool several times around the food but eventually failed to pull in the reward (Fig. 3A).
(3.12 MB MPG)

Movie S6 Level 2a, early phase. After the degu pulled the tool toward itself for a secure hold, the degu pushed the tool past the food and made two distinct movements (lateral and forward) to get the food (Fig. 3B, early).
(1.74 MB MPG)

Movie S7 Level 2b, middle phase. After the degu pulled the tool toward itself for a secure hold, the degu pushed the tool past the food made two movements as in Sv6, but these two movements occurred in quick succession (Fig. 3B, middle).
(1.49 MB MPG)

Movie S8 Level 2b, late phase. After the degu pulled the tool toward itself for a secure hold, the degu pushed the tool past the food and made a smooth clockwise movement to obtain the food (Fig. 3B, late).
(2.60 MB MPG)

Movie S9 Probe test A (contingency reversal). In trial 18, the degu was given a normal trial in which the reward was placed first, then the tool. In trial 19, the contingency was reversed and the degu kept the tool before the food was presented. The animal showed slight confusion because it did not shift its attention from the tool to the food, but eventually it succeeded in getting the food (Fig. 4-A).
(4.33 MB MPG)

Movie S10 Probe test B (generalization). In trial 32, the degu was presented with a new tool that had unfamiliar color and shape, but the animal did not hesitate to use the new tool and successfully obtained the food (Fig. 4-B).
(6.70 MB MPG)

Movie S11 Probe test C (functionality 1). Under Level 1a conditions, two tools (the familiar, functional tool, and an unfamiliar, obviously non-functional tool that lacked a blade) were presented to the degu and it selected the functional one without hesitation (Fig. 4-C).
(3.66 MB MPG)

Movie S12 Probe test D (functionality 2). Under Level 1a conditions, two tools (the familiar, function tool with the green blade and an unfamiliar, non-functional tool that had a raised non-functional, but familiar green, blade) were presented to the degu and it selected the functional one without hesitation (Fig. 4-D).
(4.25 MB MPG)

Movie S13 Probe test E (preference). The degu showed no sign of aversion to an unfamiliar but functional tool (Fig. 4-E).
(2.90 MB MPG)
